# Construction and validation of an educational booklet on prematurity for pregnant women, postpartum women and family members

**DOI:** 10.1590/0034-7167-2024-0311

**Published:** 2025-08-08

**Authors:** Bruna Vieira Oliveira, Bruna Luiza Garmatz, Stefani Araujo da Silva, Vitória da Silva Oliveira, Ana Carolina Refosco Sparremberger, Karin Viegas, Alisia Helena Weis

**Affiliations:** I Hospital Fêmina, Grupo Hospitalar Conceição. Porto Alegre, Rio Grande do Sul, Brazil; II Universidade Federal de Ciências da Saúde de Porto Alegre. Porto Alegre, Rio Grande do Sul, Brazil

**Keywords:** Validation Study, Teaching Materials, Health Education, Infant, Premature, Intensive Care Units, Neonatal, Estudio de Validación, Materiales de Enseñanza, Educación en Salud, Recien Nacido Prematuro, Unidades de Cuidado Intensivo Neonatal

## Abstract

**Objectives:**

to develop and validate an educational booklet on prematurity for pregnant women, postpartum women, and family members.

**Methods:**

a methodological study developed in three stages: preparation of the educational booklet itself; content validation by nurse judges in two stages using a Content Validity Index higher than 0.80 and an agreement rate higher than 90%; and evaluation with the target audience using the Suitability Assessment of Materials.

**Results:**

the material entitled “My Premature Baby in the Neonatal ICU: a booklet for the family” was developed, addressing topics on care and interventions for premature babies and guidance for families, validated by nurse judges with a Content Validity Index of 0.98 and evaluated with 95.39% of the variables classified as “Very good” by the target audience.

**Conclusions:**

the booklet proved to be representative, didactic, and easy to understand, contributing to the education and health promotion of caregivers of premature babies.

## RESUMEN

**Objetivos:**

desarrollar y validar una cartilla educativa sobre prematuridad para mujeres embarazadas, puérperas y familiares.

**Métodos:**

estudio metodológico desarrollado en tres etapas: elaboración del propio folleto educativo; validación de contenido por jueces de enfermería en dos etapas utilizando un Índice de Validez de Contenido mayor a 0,80 y una tasa de acuerdo mayor al 90%; y evaluación con el público objetivo utilizando la Evaluación de Idoneidad de Materiales.

**Resultados:**

construcción del material titulado “Mi bebé prematuro en la UCI Neonatal: cartilla para la familia”, abordando temas sobre cuidados e intervenciones con bebés prematuros y orientaciones para las familias, validado por enfermeros jueces con un Índice de Validez de Contenido de 0,98 y evaluado con 95,39% de las variables clasificadas como “Muy buenas” por el público objetivo.

**Conclusiones:**

la cartilla se mostró representativa, didáctica y de fácil comprensión, contribuyendo a la educación y promoción de la salud de los cuidadores de bebés prematuros.

## INTRODUCTION

Prematurity is defined as birth occurring before 37 weeks of gestation. Late preterm refers to infants born between 34 weeks and 36 weeks and 6 days, while extremely preterm applies to those born before 28 weeks^(1)^. Prematurity is associated with a high susceptibility to health issues^(2)^, particularly respiratory, cardiovascular^(3)^, motor^(4)^, and immunological disorders in newborns^(5)^. This vulnerability is closely tied to higher neonatal morbidity and mortality rates, which can often be mitigated through low-cost interventions such as adequate prenatal care, neonatal support, and health education initiatives^(1)^.

A report published in 2023 by the World Health Organization and the United Nations Children’s Fund indicates that, among the 13.4 million preterm births recorded globally in 2020, nearly one million resulted in infant death^(6)^. Brazil ranks ninth worldwide in absolute numbers of preterm births^(7)^. This scenario presents significant challenges for healthcare services and adds complexity to the lives of families with preterm newborns, who often face uncertainty and insecurity about this condition and the neonatal intensive care unit (NICU) environment. Family members frequently report feelings of fear, helplessness, powerlessness, guilt, and stress, with physical and psychological health issues being particularly prevalent among mothers^(8-10)^. This reality underscores the need for emotional support and appropriate guidance to mitigate these impacts on pregnant women, postpartum women, and families.

The critical role of health education as a cornerstone of care during prematurity is widely supported by research. As a strategy to reduce stress and empower caregivers, health education fosters active family participation in care and helps develop knowledge to better support preterm infants^(11-13)^. For instance, educational booklets serve as practical tools to assist families by providing relevant information in accessible and clear language^(8)^, enhancing understanding of the NICU environment and necessary care practices^(8,14)^. Additionally, healthcare professionals play an essential role in creating these materials, contributing not only to the dissemination of technical information but also to improving the emotional well-being of families^(15,16)^.

Thus, democratizing access to scientifically validated educational materials, such as booklets, is imperative. These resources enhance families’ understanding of the NICU environment and care processes while providing much-needed guidance and support.

## OBJECTIVES

To develop and validate an educational booklet on prematurity for pregnant women, postpartum women, and their families.

## METHODS

### Ethical aspects

This study adhered to Resolution No. 466/2012 of the National Health Council, complying with all ethical standards for research involving human participants^(17)^. It was approved by the Research Ethics Committee of the Federal University of Health Sciences of Porto Alegre (CEP-UFCSPA in portuguese).

### Study Design, Period, and Location

This methodological study encompassed three processes: development, production, and construction of tools; validation of tools; and evaluation and/or application of tools, aimed at improving research or care practices^(18)^. Following the methodological study steps, the booklet was produced, its content was validated by nurse judges, and it was evaluated by the target audience between July 2021 and February 2023 in a hospital in southern Brazil. The article was organized using the Consolidated Criteria for Reporting Qualitative Research (COREQ) checklist^(19)^.

### Population or sample; inclusion and exclusion criteria

The committee of experts responsible for validating the content and appearance of the booklet was composed of a convenience sample. The recommended sample size for validation, as indicated in the literature, ranges from 5 to 10 judges^(20,21)^. These experts were required to meet at least two of the criteria described by Jasper^(22)^: a minimum of five years of professional experience (in clinical practice and/or research), technoscientific production in the maternal-child area, and academic qualifications as a specialist, master, or doctor. In both stages, 10 national nurses selected via the Lattes curriculum database participated. Participants on legal leave during data collection were excluded.

For the target audience evaluation, 30 participants were included, comprising hospitalized pregnant women at risk of preterm birth, postpartum women, and/or family members of infants admitted to the NICU of a hospital in southern Brazil. Participants experiencing emotional instability at the time of the intervention were excluded.

### Study Protocol

The first version of the booklet was developed based on 20-minute semi-structured interviews with five professionals (a physiotherapist, nurse, physician, speech therapist, and pharmacist) from a neonatal service at a hospital in southern Brazil, due to their direct involvement in the care of preterm infants and their families. The following guiding questions were used during the interviews: “1) What topics do you believe are pertinent to address with women and their families who may have their baby admitted to the NICU? 2) What aspects of care do you consider important to describe regarding prematurity and potential interventions that newborns may receive during their stay in the NICU? 3) What care do you believe is essential to discuss with pregnant women during hospitalization due to pregnancy complications related to prematurity? 4) What guidance about the intensive care environment should be included in the educational material for pregnant women and their families?”. Additionally, a literature review on prematurity aided in defining the content.

With the collaboration of a graphic designer, the booklet’s artwork was created-including illustrations, formatting, configuration, and page layout-marking the completion of the material’s construction phase. To adapt the language used in the booklet, the researcher applied the Clear Communication Index (CDC) guidelines^(23)^. These guidelines were implemented by incorporating communication resources such as questions and answers, fragmented text, main titles and headers, boxes, attention-grabbing phrases, active voice, everyday language, bullet points, lists, tables, block organization, illustrations, and photos.

In the second stage, the booklet underwent content and appearance validation by nurse judges. Specialists were invited to participate via email. Those who agreed to contribute received a link to an electronic form (Google Forms), and consent to participate in the study was a prerequisite for accessing subsequent pages of the questionnaire.

In the first validation round, five judges participated^(21)^. They assessed the items in each section-determining their scope, whether each section was adequately represented by its set of items, and if all aspects were addressed. They also evaluated whether the content was appropriate, representative, and well structured. A concordance rate greater than 90% was applied to the items^(24)^, and suggestions for the inclusion or elimination of items were allowed^(24)^.

In the second validation round, five nurse judges individually evaluated each item, including the booklet’s format, title, instructions, and the clarity and relevance of each aspect to be assessed. For clarity, they analyzed whether the wording of the items was comprehensible and specifically expressed the intended meaning. Regarding relevance or representativeness, they verified whether the items accurately reflected the concepts involved, were relevant, and met the proposed objectives. This phase achieved a Content Validity Index (CVI) greater than 0.8, as recommended in the literature^(24)^. Judges could also provide suggestions and/or comments^(25)^.

The third stage involved evaluating the booklet’s comprehension by its target audience using the Suitability Assessment of Materials (SAM) instrument^(26)^. SAM consists of 21 items scored from zero to two, classified as follows: (0) Inadequate; (1) Adequate; (2) Superior. This instrument assesses educational materials for patients, enriching professional-patient communication and promoting more effective interactions^(26)^. Participants were contacted by the research team in the maternal-infant service and approached in a private environment to ensure comfort. They received printed copies of the booklet, were guided on critical reading, and subsequently responded to the researchers’ questions using the SAM instrument.

### Analysis of Results and Statistics

During the booklet construction phase, semi-structured interviews were recorded and transcribed. Data analysis was performed using thematic content analysis^(27)^. Data were entered into Microsoft Office Excel for subsequent organization into tables and charts.

The booklet’s content analysis included evaluating the agreement rates among judges, with a minimum acceptable agreement rate of 90%^(24)^. Items with lower agreement rates were revised or rewritten based on the evaluations, resulting in a second version of the booklet. Additionally, the CVI was calculated, with values above 0.80 considered acceptable^(28)^, indicating strong validity.

Items with CVI scores below the established threshold were adjusted accordingly^(20)^. The booklet’s comprehension was evaluated by calculating the total scores divided by the total number of SAM items. Classifications were as follows: “Superior” (100%) and “Adequate” (80-99.9%). Below this range, the booklet would need to be reformulated for the target audience and re-evaluated^(26)^.

## RESULTS

### Booklet Development

During the development phase, the NICU’s multidisciplinary team was interviewed. The analysis of these interviews identified themes for the booklet, covering topics such as the NICU environment, care timelines, hospital discharge, comprehensive support, preterm infant care and breastfeeding, and considerations regarding the mental health of the preterm infant’s family. Based on the defined topics, content, and formatting, the first version of the booklet was created and subsequently submitted for validation.

### Validation by Judges

In the first validation round, five judges-all female nurses, predominantly aged 30-39 years (60%), with postgraduate specialization as their highest academic qualification (60%) and 5-10 years of patient care experience (60%)-participated. The booklet was divided into 11 domains for validation. Most domains achieved an agreement rate above 90%^(24)^ and required no changes. However, some domains, despite meeting the agreement threshold, were revised based on relevant suggestions to improve the product.

One item, “Getting to Know My Preterm Baby”, did not achieve the required 90% agreement^(24)^ during the first round. Responses to the questions “What can I expect from my preterm baby?” and “What is prematurity?” were revised due to the use of technical terms that judges felt might hinder understanding by the target audience-parents and families of preterm infants. Additional changes included incorporating non-heteronormative language by replacing titles like “dad” and “mom” with “parent(s)” to ensure inclusivity. Moreover, the phrase “current literature” in the booklet’s introduction was replaced with “best health evidence” to clarify the source of information (Table 1).

**Table 1:** Agreement Across Rounds for Content Validation, Degree of Agreement, and Content Validity Index, Porto Alegre, Rio Grande do Sul, Brazil, 2023

Item	First round - Degree of Agreement (%)		Second round - Content Validity Index (CVI)	
	Representativeness	Clarity	Representativeness	Clarity
Domain 1 - Title, presentation and summary				
1.1 Title	100	100	1	1
1.2 Summary	100	100	1	1
1.3 Presentation	100	100	1	1
Domain 2 - The NICU^*^				
2.1 Items found in the NICU^*^	100	100	1	1
2.2 Paragraph on the NICU^*^	100	100	1	1
Domain 3 - The NICU^*^ environment				
3.1 Paragraph on the NICU^*^ environment	100	100	1	1
3.2 Item "Lighting"	100	100	1	1
3.3 Item "Care for parents"	100	100	1	1
3.4 Item "Sounds and noises"	100	100	1	1
3.5 Item "Baby care"	100	100	1	0.8
Domain 4 - Getting to know my premature baby				
4.1 Item "What is prematurity?"	100	80	1	1
4.2 Item "What to expect from a premature baby"	100	60	1	1
Domain 5 - Babies and their development				
5.1 Item "Babies and their development"	100	100	1	0.6
5.2 Item "That's why you'll often hear about corrected gestational age	100	100	1	1
Domain 6 - My baby needed to stay in the NICU^*^, what do I need to know?				
6.1 Item "Help with breathing"	100	100	1	1
6.2 Item "Campanula"	100	100	1	1
6.3 Item "CPAP^**^"	100	100	1	0.8
6.4 Item "Respirator"	100	100	1	1
6.5 Item "Help with feeding"	100	100	1	1
6.6 Item "Did you know"	100	100	1	0.8
6.7 Item "Fun fact"	100	100	1	1
6.8 Item "Type of feeding"	100	100	1	1
6.9 Item "Help with maintaining my temperature"	100	100	1	0.8
6.10 Item "Discharge of the premature baby"	100	100	1	1
Domain 7 - Breastfeeding my premature baby				
7.1 Item "Breast milk"	100	100	1	1
7.2 Item "Colostrum therapy"	100	100	1	1
7.3 Item "Milk production maternal"	100	100	1	1
7.4 Item "Extracting breast milk"	100	100	1	1
7.5 Item "Position, offering and breastfeeding of the newborn"	100	100	1	1
7.6 Item "Nipples and bottles"	100	100	0.8	0.8
7.7 Item "Breastfeeding devices"	100	100	1	1
Domain 8 - Care while my baby is in the NICU^*^				
8.1 Item "Care while my baby is in the NICU^*^	100	100	1	1
Domain 9 - Mom and Dad, relax!				
9.1 Item "Mom and Dad, relax!	100	100	1	1
Domain 10 - Useful phone numbers and websites				
10.1 Item "Useful phone numbers and websites"	100	100	1	1
Domain 11 - References				
11.1 Item "References"	100	100	1	1

* NICU - neonatal intensive care unit.

** CPAP - continuous positive airway pressure.

* *ICU - intensive care unit.*

In the second validation round, five judges-all female nurses, mostly aged 30-39 years (60%), with master’s degrees as the most common academic qualification (40%), and 10-20 years of experience (60%)-participated. They evaluated the booklet as a whole, including its title, format (layout), and individual items, assessing clarity and representativeness using a Likert scale from 1 to 4. All 11 domains were maintained and evaluated for clarity and representativeness in the second round (Table 1).

During the second round of content validation, the judges did not achieve a CVI greater than 0.8 for the item “Babies and Their Development”. The justification was that the illustration needed modification because it could instill fear in the parents of preterm infants and its meaning was not well understood. Consequently, it was decided to exclude it from the booklet.

Furthermore, even with adequate agreement rates and CVI, additional edits were made based on the judges’ suggestions. These included changing the font color to improve readability and modifying or adding words to enhance the specificity of the items and facilitate comprehension for parents. The final validation by the judges reached an overall CVI of 0.98. Subsequently, the booklet was submitted for validation by the target audience.

### Validation by the target audience

For this stage, 30 participants were selected, of whom 28 (93.33%) were female and 19 (63.33%) were between 30 and 39 years old. All participants had completed elementary education, and 13 (43.33%) reported having no knowledge of prematurity.

Items related to content, literacy level, illustrations, layout, stimulation, motivation, learning, and cultural appropriateness were validated by the target audience, as all achieved an approval rate of 80% or higher. The overall evaluation of the booklet yielded very positive results, with 95.39% of the variables rated as “Very Good”, while the disapproval rate was 0.95%. Most of the items deemed inadequate were related to the text’s font formatting (Table 2).

**Table 2:** Booklet Evaluation: Validation Stage with the Target Audience, Porto Alegre, Rio Grande do Sul, Brazil, 2023

Variables	Very good (2) n (%)	Adequate (1) n (%)	Inadequate (0) n (%)
1 Content			
1.1 Regarding the content of the booklet, what do you think?	30 (100)	-	-
1.2 Do you understand the purpose of the material (does it provide information about prematurity and the baby's hospitalization)?	30 (100)	-	-
1.3 Does the booklet have its objective clearly stated in the title and presentation?	29 (96.67)	1 (3.33)	-
1.4 Was the booklet useful in providing information about prematurity and the baby's hospitalization in the NICU^*^?	29 (96.67)	1 (3.33)	-
1.5 Does the booklet and its content meet the objective (to assist with information about the NICU^*^)?	29 (96.67)	1 (3.33)	-
1.6 Does the booklet present key points, examples and illustrations to better understand the text?	30 (100)	-	-
2 Literacy level			
2.1 Who read the booklet? (did you read it alone (2)/in some parts you needed help (1)/did someone read it to you (0))	29 (96.67)	1 (3.33)	-
2.2 About the writing of the booklet?	29 (96.67)	1 (3.33)	-
2.3 Is there a context in the booklet before the information?	28 (93.33)	2 (6.67)	-
2.4 Are the words used in the booklet easy to understand?	29 (96.67)	1 (3.33)	-
2.5 Are there headings or captions that briefly inform what comes next?	28 (93.33)	1 (3.33)	1 (3.33)
3 Illustrations			
3.1 Regarding the illustrations, do you think:	28 (93.33)	2 (6.67)	-
3.2 Are the illustrations related to the content covered?	30 (100)	-	-
4 Layout			
4.1 Are there subtitles in the booklet?	30 (100)	-	-
4.2 Regarding the size of the lettering in the booklet?	24 (80)	5 (16.67)	1 (3.33)
4.3 Regarding the lettering in the booklet?	25 (83.33)	3 (10)	2 (6.67)
4.4 Did you identify in the booklet: -The use of colors does not distract from the content; -That the illustrations are on the same page as the related text; -The layout and order of the information present a logical sequence; - Resources such as shading, boxes, and arrows are used to direct attention to important points; - The pages are ordered.	30 (100)	-	-
5 Stimulation, motivation, and learning			
5.1 Did the booklet motivate you to deal with prematurity and help you with attitudes and behaviors?	27 (90)	2 (6.67)	1 (3.33)
5.2 Do you believe that the information contained in the booklet can be put into practice - useful?	30 (100)	-	-
6 Cultural suitability			
6.1 Does the booklet help with your experience with prematurity?	30 (100)	-	-
6.2 Does the booklet address situations similar to those you are experiencing?	27 (90)	2 (6.67)	1 (3.33)

* *NICU - neonatal intensive care unit.*

**Figure 1:**
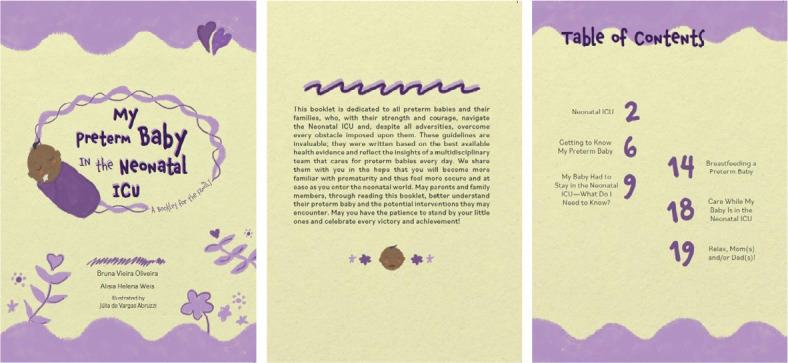
Cover, presentation and summary of the educational booklet on prematurity, Porto Alegre, Rio Grande do Sul, Brazil, 2023

Once the validation and adjustment stages were completed, the final version of the material was produced. Thus, the educational booklet on prematurity, titled “My Preterm Baby from the Neonatal ICU: A Booklet for the Family”, consists of 21 pages, including a cover, with standard dimensions of 21 cm in height by 15 cm in width, and is presented in lilac and yellow. The content is organized into the following topics: The Neonatal ICU; Getting to Know My Preterm Baby; My Baby Had to Stay in the Neonatal ICU-What Do I Need to Know?; Breastfeeding a Preterm Baby; Care While My Baby Is in the Neonatal ICU; and Relax, Mom(s) and/or Dad(s)! (Figure 1).

## DISCUSSION

Health professionals, especially nurses, have fostered creativity in the process of caring and educating by employing a diverse array of educational technologies^(29)^. These technologies should be used in ways that actively promote the inclusion of individuals in the educational context, strengthening the values of citizenship and autonomy among participants^(30)^, assisting in teaching-learning processes, and enhancing health education initiatives^(31)^. Among the educational materials available in the literature-such as informational and educational resources-the use of educational booklets stands out^(32-35)^.

As an educational technology, the production of booklets plays an important role in forming knowledge and developing the skills of patients and/or their families^(36)^. This material contributes to the construction of technical-scientific knowledge through an integrated approach that combines the experiences of professionals and users, establishes systematic practices, mediates the educational process, and complements the guidance provided during health care appointments^(37-39)^, thereby redefining the experience of neonatal hospitalization for the Family.

To convey information effectively, careful attention must be paid to word choice by using practical vocabulary for the target audience and creating clear, easily understandable text^(40)^. In addition, the use of colors, illustrations, and boldface text makes the work more dynamic, attractive, and inviting to read^(41)^. The prematurity booklet stood out by featuring real photos that depict the preterm baby’s journey through the NICU, providing families with a first contact with the environment and routines in a more welcoming and accessible manner. These elements are important for sensitizing families and sparking interest in reading the material^(42)^.

Studies that have developed educational materials on prematurity have reported positive results, as they contributed significantly to the construction of knowledge and increased mothers’ confidence in caring for their children^(43)^. Moreover, they promoted the empowerment of caregivers to continue providing care and to prepare for their children’s hospital discharge^(32)^. These results corroborate the importance of developing educational technologies that support health education initiatives for families, especially in high-complexity settings such as neonatal care^(32)^.

In this regard, the literature presents a variety of technologies widely used in health education by nursing-from traditional methods to innovative approaches such as online courses, workshops, discussion circles, classes, case discussions, collaborative platforms, and didactic materials. Such technologies play a fundamental role in promoting family-centered care^(44)^.

Consequently, health education initiatives mediated by the educational booklet on prematurity serve as an essential tool for professionals-especially nurses-in delivering family-centered care, yielding practical outcomes in the care and understanding of preterm infant health. Moreover, the booklet facilitates dialogue and the creation of bonds with families, particularly during times of great emotional vulnerability, representing an advance in the quality of nursing care.

It is important to note that, in this study, the material was subjected to validation by specialists in the field, ensuring its technical adequacy as an essential step. The specialist validation phase is fundamental to guaranteeing the scientific foundation of the presented content. It is imperative that the instruments used to validate educational materials in health can reliably cover any subject^(45,46)^. Thus, the validation carried out by nurse judges, in terms of both content and visual representation, brought several improvements to the prematurity booklet, aligning it more closely with the experiences of families with a preterm baby in the NICU. In addition, validation with the target audience is necessary to ensure that the resource meets specific needs and is effective in supporting health education initiatives. In this regard, another study that developed and validated a booklet on fall prevention in hospitals, using specialists, evaluated the adequacy of the educational material with patients using the SAM. This strategic combination makes the material more robust and faithful to the individuals’ context^(42)^.

The educational booklet validated in this study achieved an overall CVI of 0.98 by the judges, and according to the target audience, 95.39% of the variables were rated as “Very Good”, corroborating literature findings that demonstrate the relevance of the topic discussed and the efficiency of the technology used in conveying the information^(6,42,47)^. Moreover, the material was evaluated within its thematic context by specialists in the field of interest, underscoring the validity of its content.

It is noteworthy that this booklet is distinct because it is designed both for preparation and for the period of hospitalization in the neonatal unit, adopting a sensitive and welcoming perspective for pregnant women, postpartum women, and families^(42)^. It has the potential to be applied at various levels of healthcare in maternal-infant services, whether in Primary Health Care during high-risk prenatal care for preterm birth or in the NICU of hospitals, assisting in providing guidance and clarifying doubts during high-risk pregnancies, premature labor, and preterm birth.

### Study limitations

One limiting factor of this study was that the survey of topics on prematurity was conducted solely with health professionals working in the NICU. The contribution of users and professionals from other levels of healthcare, such as Primary Health Care-which oversees prenatal care for pregnancies at risk of preterm birth-would have enriched the discussion of potential topics for constructing the booklet by offering a different perspective on the care of pregnant women.

### Contributions to the field of health

The development of an educational material that addresses risk factors for prematurity and the characteristics of preterm newborns, intended for pregnant women and families of preterm newborns admitted to a NICU, may help promote health and prevent adverse outcomes for neonates. Moreover, it will aid in understanding this phase of fear and uncertainty by providing clear, emotionally supportive information to guide families in facing the challenges of prematurity. Additionally, it serves as a facilitating tool that can be used by health professionals-especially nurses-to deliver clinical information and guidance to the families of newborns. This contributes to the preparation for and management of the adversities associated with prematurity, ensuring an improvement in the quality of healthcare in the maternal-infant field.

## CONCLUSIONS

The construction and validation of a health education technology in the form of a booklet aims to prepare families for the potential outcomes of prematurity while serving as a facilitating tool for improving the quality of nursing care. The participation of nurse judges contributed to the refinement and enhancement of the material, which was finalized with an overall CVI of 0.98. Additionally, the target audience’s participation, with an approval rating of 95.39%, demonstrated significance and confirmed that the proposed objectives were met. The process was rigorously followed, resulting in a representative, didactic, and easily understandable material that expands opportunities for education and health promotion among caregivers of preterm newborns.

## Data Availability

Not applicable.
