# Supplementary material for: Construction and validation of an educational booklet on prematurity for pregnant women, postpartum women and family members
Source: Rev Bras Enferm. 2025 Aug 8;78(Suppl 2):e20240311. doi: 10.1590/0034-7167-2024-0311 (PMC12334137; doi:10.1590/0034-7167-2024-0311)
Supplement: Translation

# Translation: Construção e validação de cartilha educativa sobre prematuridade para gestantes, puérperas e familiares

**DOI:** 10.1590/0034-7167-2024-0311pt

**Authors:** Bruna Vieira Oliveira, Bruna Luiza Garmatz, Stefani Araujo da Silva, Vitória da Silva Oliveira, Ana Carolina Refosco Sparremberger, Karin Viegas, Alisia Helena Weis

**Affiliations:** I Hospital Fêmina, Grupo Hospitalar Conceição. Porto Alegre, Rio Grande do Sul, Brasil; II Universidade Federal de Ciências da Saúde de Porto Alegre. Porto Alegre, Rio Grande do Sul, Brasil

**Keywords:** Estudo de Validação, Materiais de Ensino, Educação em Saúde, Recém-Nascido Prematuro, Unidades de Terapia Intensiva Neonatal

## Abstract

**Objetivos:**

desenvolver e validar uma cartilha educativa sobre prematuridade para gestantes, puérperas e familiares.

**Métodos:**

estudo metodológico desenvolvido em três etapas: elaboração da cartilha educativa; validação de conteúdo por juízes enfermeiros em duas etapas, por meio do Índice de Validade de Conteúdo superior a 0,80 e taxa de concordância maior que 90%; e avaliação com o público-alvo usando o *Suitability Assessment of Materials*.

**Resultados:**

construção do material intitulado “Meu bebê prematuro na UTI Neonatal: cartilha para a família”, que aborda temas sobre os cuidados e intervenções com o bebê prematuro, além de orientações às famílias. O material foi validado por juízes enfermeiros, com Índice de Validade de Conteúdo de 0,98, e avaliado com 95,39% das variáveis classificadas como “Muito bom” pelo público-alvo.

**Conclusões:**

a cartilha mostrou-se representativa, didática e de fácil compreensão, contribuindo para a educação e promoção da saúde de cuidadores de bebês prematuros.

## INTRODUÇÃO

A prematuridade consiste no nascimento antes das 37 semanas de gestação. São classificados como tardios os nascidos entre 34 semanas e 36 semanas e 6 dias, e como prematuros extremos aqueles nascidos antes de 28 semanas^(1)^. Ela se caracteriza pela alta suscetibilidade ao desenvolvimento de problemas de saúde^(2)^, envolvendo principalmente distúrbios respiratórios, cardiovasculares^(3)^, motores^(4)^ e imunológicos em recém-nascidos (RN)^(5)^. Essa vulnerabilidade está intimamente ligada a uma maior taxa de morbidade e mortalidade neonatal, podendo ser reduzida com intervenções de baixo custo, como cuidados pré-natais adequados, suporte neonatal e ações de educação em saúde^(1)^.

O relatório publicado em 2023 pela Organização Mundial da Saúde e pelo Fundo das Nações Unidas para a Infância indica que, dentre os 13,4 milhões de partos prematuros registrados em 2020 no mundo, quase um milhão resultou no óbito do bebê^(6)^. No contexto global, o Brasil representa o nono país em número absoluto de prematuros^(7)^. Assim, o cenário atual representa um desafio para os serviços de saúde e uma complexidade adicional para as famílias dos RN, que muitas vezes se deparam com o desconhecimento e inseguranças relacionadas a essa condição e ao ambiente da unidade de terapia intensiva neonatal (UTIN). Os familiares frequentemente sentem medo, desamparo, impotência, culpa e estresse, podendo apresentar problemas de saúde física e psicológica, especialmente as mães^(8-10)^. Tal realidade reforça a necessidade de fornecer suporte emocional e orientação adequada para mitigar esses impactos nas gestantes, puérperas e familiares.

A relevância da educação em saúde, como alicerce do cuidado durante a prematuridade, é amplamente respaldada pela pesquisa. Como estratégia para a redução do estresse e o empoderamento dos cuidadores, ela contribui para a participação ativa das famílias no cuidado e na construção de conhecimentos para o melhor desenvolvimento dos bebês prematuros^(11-13)^. A título de exemplo, materiais educativos do tipo cartilha podem ser considerados tecnologias educativas práticas para auxiliar as famílias, fornecendo informações relevantes em uma linguagem acessível e clara^(8)^, ampliando o entendimento sobre o ambiente da UTIN e os cuidados necessários^(8,14)^. Além disso, os profissionais da saúde são essenciais na elaboração desses materiais, pois desempenham um papel crucial não apenas na transmissão de informações técnicas, mas também na melhoria do bem-estar emocional das famílias^(15,16)^.

Dessa forma, a democratização do acesso a materiais educativos, como cartilhas validadas cientificamente, torna-se imperativa, pois esses recursos são valiosos para ampliar a compreensão das famílias sobre o ambiente e os cuidados prestados na UTIN, ao mesmo tempo que oferecem suporte e orientação.

## OBJETIVOS

Desenvolver e validar uma cartilha educativa sobre prematuridade para gestantes, puérperas e familiares.

## MÉTODOS

### Aspectos éticos

Esta pesquisa seguiu a Resolução do Conselho Nacional de Saúde nº 466/2012, cumprindo todas as normas éticas para o desenvolvimento de pesquisas que envolvem seres humanos^(17)^. Ela foi aprovada pelo Comitê de Ética em Pesquisa da Universidade Federal de Ciências da Saúde de Porto Alegre (CEP-UFCSPA).

### Desenho, período e local de estudo

Trata-se de um estudo metodológico que compreende três processos: desenvolvimento, produção e construção de ferramentas; validação de ferramentas; e avaliação e/ou aplicação de ferramentas, aprimorando a pesquisa ou a prática assistencial^(18)^. Seguindo as etapas do estudo metodológico, foi realizada a produção da cartilha, a validação do conteúdo por juízes enfermeiros, bem como sua avaliação pelo público-alvo, entre julho de 2021 e fevereiro de 2023, em um hospital do sul do Brasil. Para a organização do artigo, foi utilizado o *checklist Consolidated Criteria for Reporting Qualitative Research (COREQ)* ^(19)^.

### População ou amostra; critérios de inclusão e exclusão

O comitê de especialistas responsável pela validação do conteúdo e aparência da cartilha foi composto por uma amostra por conveniência. A amostra necessária para validação, conforme indicado pela literatura, foi de 5 a 10 juízes^(20,21)^. Eles deveriam atender pelo menos dois requisitos descritos por Jasper^(22)^: experiência profissional mínima de 5 anos (clínica e/ou pesquisa), produção tecnocientífica na área materno-infantil e titulação acadêmica de especialista, mestre ou doutor. Nas duas etapas, participaram 10 enfermeiros nacionais selecionados pelo currículo Lattes. Foram excluídos os participantes que estavam em afastamento legal no momento da coleta.

Para a avaliação do público-alvo, foram incluídas 30 participantes, sendo elas gestantes internadas com risco de parto prematuro, puérperas e/ou familiares de bebês internados na UTIN de um hospital do sul do Brasil. Foram excluídas as participantes que se encontravam em condições de desequilíbrio emocional no momento da intervenção.

### Protocolo de estudo

A primeira versão da cartilha surgiu a partir de entrevistas semiestruturadas com duração de 20 minutos, realizadas com cinco profissionais (fisioterapeuta, enfermeiro, médico, fonoaudiólogo e farmacêutico) de um serviço neonatal em um hospital do sul do Brasil, devido ao seu envolvimento direto no cuidado aos bebês prematuros e suas famílias. Para a realização das entrevistas, foram utilizadas as seguintes perguntas norteadoras: “1) Quais temas você acredita serem pertinentes para abordar com mulheres e seus familiares que possivelmente terão seu bebê internado na UTI Neonatal? 2) Quais cuidados você considera pertinentes para serem descritos em relação à prematuridade e às possíveis intervenções que os recém-nascidos podem receber durante a internação na UTI Neonatal? 3) Quais cuidados você acredita serem importantes abordar com as gestantes durante sua internação devido a intercorrências na gestação relacionadas à prematuridade? 4) Quais orientações relacionadas ao ambiente intensivo de cuidado devem ser incluídas no material educativo para gestantes e seus familiares?”.

Com a colaboração de um designer gráfico, desenvolveu-se a arte da cartilha por meio da criação de ilustrações e da formatação, configuração e diagramação das páginas, marcando o término da fase de construção do material. Para a adequação da linguagem utilizada na cartilha, foram adotadas pela pesquisadora as diretrizes do *Clear Communication Index* (CDC)^(23)^ e aplicadas na construção da cartilha, com o uso de recursos de comunicação, como a utilização de perguntas e respostas, textos fragmentados, uso do título principal e cabeçalhos, caixas, frases que chamam a atenção, uso da voz ativa, palavras de uso cotidiano, uso de tópicos, listas, tabelas, organização em seções, ilustrações e fotos.

Na segunda etapa, a cartilha foi submetida à validação de conteúdo e aparência pelos juízes enfermeiros. O convite aos especialistas foi feito via e-mail. Em sequência, àqueles que sinalizaram aceitar participar da validação, foi encaminhado o link de acesso ao formulário eletrônico (*Google Forms*), sendo que a concordância em participar do estudo era uma condição essencial para acessar as páginas seguintes do questionário.

Na primeira rodada de validação, cinco juízes participaram^(21)^. Eles avaliaram os itens de cada seção, determinando sua abrangência, se cada seção estava adequadamente representada por seu conjunto de itens e se todos os aspectos foram abordados. Além disso, verificaram se o conteúdo era adequado, representativo e bem estruturado. Foi utilizada uma taxa de concordância superior a 90% nos itens^(24)^, e sugestões quanto à inclusão ou à eliminação de itens puderam ser realizadas^(24)^.

Na segunda rodada de validação, cinco juízes enfermeiros avaliaram cada item individualmente, considerando o formato do instrumento, o título, as instruções e a clareza e pertinência de cada aspecto a ser avaliado. Com relação à clareza, analisaram se a redação dos itens era compreensível e expressava especificamente o significado pretendido. Quanto à pertinência ou representatividade, verificaram se os itens refletiam com precisão os conceitos envolvidos, eram relevantes e adequados para atingir os objetivos propostos, culminando em um Índice de Validade de Conteúdo superior a 0,8, conforme recomendado na literatura^(24)^. Os juízes também puderam fazer sugestões e/ou comentários^(25)^.

A terceira etapa consistiu na avaliação da compreensão da cartilha pelos usuários. Foi utilizado o instrumento *Suitability Assessment of Materials* (SAM)^(26)^, composto por 21 itens pontuados de zero a dois, classificados da seguinte forma: (0) Inadequado; (1) Adequado; (2) Muito bom. Esse instrumento permite avaliar os materiais educativos para pacientes, enriquecendo o processo de comunicação e interação profissional-paciente e proporcionando uma comunicação mais eficaz^(26)^. Os participantes dessa etapa foram contatados pela equipe de pesquisadores no serviço materno-infantil e abordados em um ambiente reservado, garantindo conforto durante o contato. Eles receberam a cartilha impressa e foram orientados sobre a leitura crítica da mesma; na sequência, responderam aos questionamentos dos pesquisadores com o SAM.

### Análise dos resultados e estatística

Na etapa de construção da cartilha, as entrevistas semiestruturadas foram gravadas e transcritas, e a análise de dados foi realizada utilizando a técnica de análise de conteúdo do tipo temática^(27)^. Os dados foram inseridos no software *Microsoft Office Excel* para posterior organização em tabelas e quadros.

A análise de conteúdo da cartilha incluiu a avaliação das Taxas de Concordância entre os juízes, estabelecendo como padrões razoáveis um mínimo de 90% de concordância^(24)^. Os itens com concordância abaixo desse percentual foram reelaborados ou reescritos, de acordo com a avaliação, originando uma segunda versão da cartilha. Paralelamente, foi calculado o Índice de Validade de Conteúdo (IVC), considerando como aceitáveis valores superiores a 0,80^(28)^, indicativos de uma boa validade.

Diante dos resultados, procedeu-se ao ajuste dos itens que atingiram valores inferiores ao IVC previsto no estudo^(20)^. A avaliação da compreensão da cartilha foi realizada a partir do somatório total dos escores, dividido pelo número total de itens do questionário SAM. A classificação é considerada ‘superior’ (100%) e ‘adequada’ (de 80 a 99,9%). Abaixo desse valor, a cartilha deveria ser reformulada para o público-alvo e reavaliada em novo processo de avaliação^(26)^.

## RESULTADOS

### Elaboração da cartilha

Na etapa de elaboração da cartilha, a equipe multiprofissional da UTIN foi entrevistada. A análise dessas entrevistas permitiu a identificação dos temas para a composição da cartilha, englobando tópicos como o Ambiente na UTI Neonatal, o Tempo no Cuidado, Alta Hospitalar, o Suporte Integral, Cuidados e Amamentação do Bebê Prematuro e Considerações sobre a Saúde Mental da Família do Bebê Prematuro. Com os tópicos, conteúdos e formatação definidos, a primeira versão da cartilha foi elaborada. Na sequência, o material foi submetido ao processo de validação.

### Validação por juízes

Na primeira rodada de validação da cartilha, participaram cinco juízes, todos do sexo feminino, enfermeiros, em sua maioria na faixa etária de 30-39 anos (60%), com pós-graduação - especialização como formação acadêmica máxima (60%) e atuando na assistência ao paciente por um período de 5 a 10 anos (60%). Para a validação, a cartilha foi dividida em 11 domínios. Grande parte dos domínios teve seu grau de concordância superior a 90%^(24)^, não necessitando de modificações. Contudo, alguns domínios, mesmo com grau de concordância superior a 0,9, foram reformulados, considerando as sugestões pertinentes para a melhoria do produto.

Os juízes, na primeira rodada, não alcançaram um nível de concordância superior a 90%^(24)^ no item “Conhecendo meu bebê prematuro”. As respostas às duas perguntas: “O que esperar do meu bebê prematuro?” e “O que é prematuridade?” precisaram ser reformuladas devido ao uso de termos técnicos que, na visão dos juízes, poderiam dificultar o entendimento da população-alvo - pais e familiares de bebês prematuros. Além disso, outras sugestões foram atendidas, como a inclusão de casais que não seguem o padrão heteronormativo, realizando a mudança de títulos, onde somente as palavras “papai” e “mamãe” foram utilizadas, para as palavras “papai(s)” e/ou “mamãe(s)”, bem como a substituição da expressão “literatura atual” na apresentação da cartilha por “melhores evidências de saúde”, ao explicar de onde foram retiradas as informações contidas na cartilha, no domínio “Apresentação” (Tabela 1).

**Tabela 1:** Concordância das rodadas para validação de conteúdo, Grau de Concordância e Índice de Validade de Conteúdo, Porto Alegre, Rio Grande do Sul, Brasil, 2023

Item	Primeira rodada - Grau de Concordância (%)		Segunda rodada - Índice de Validade de Conteúdo (IVC)	
	Representatividade	Clareza	Representatividade	Clareza
Domínio 1 - Título, apresentação e sumário				
1.1 Título	100	100	1	1
1.2 Sumário	100	100	1	1
1.3 Apresentação	100	100	1	1
Domínio 2 - A UTI^*^ Neonatal				
2.1 Itens encontrados na UTI^*^ Neonatal	100	100	1	1
2.2 Parágrafo sobre UTI^*^ Neonatal	100	100	1	1
Domínio 3 - O ambiente da UTI^*^ Neonatal				
3.1 Parágrafo sobre o ambiente da UTI^*^ Neonatal	100	100	1	1
3.2 Item “Iluminação”	100	100	1	1
3.3 Item “Cuidado para os pais”	100	100	1	1
3.4 Item “Sons e ruídos”	100	100	1	1
3.5 Item “Cuidados com o bebê”	100	100	1	0,8
Domínio 4 - Conhecendo meu bebê prematuro				
4.1 Item “O que é a prematuridade?”	100	80	1	1
4.2 Item “O que esperar de um bebê prematuro”	100	60	1	1
Domínio 5 - Bebês e seu desenvolvimento				
5.1 Item “Bebês e seu desenvolvimento”	100	100	1	0,6
5.2 Item “Por isso, muitas vezes, você irá ouvir sobre idade gestacional corrigida	100	100	1	1
Domínio 6 - Meu bebê precisou ficar na UTI^*^ Neonatal, o que preciso saber?				
6.1 Item “Ajuda para respirar”	100	100	1	1
6.2 Item “Campânula”	100	100	1	1
6.3 Item “CPAP^**^”	100	100	1	0,8
6.4 Item “Respirador”	100	100	1	1
6.5 Item “Ajuda para me alimentar”	100	100	1	1
6.6 Item “Você sabia”	100	100	1	0,8
6.7 Item “Curiosidade”	100	100	1	1
6.8 Item “Tipo de alimentação”	100	100	1	1
6.9 Item “Ajuda para manter minha temperatura”	100	100	1	0,8
6.10 Item “Alta do bebê prematuro”	100	100	1	1
Domínio 7 - Amamentando meu bebê prematuro				
7.1 Item “Leite materno”	100	100	1	1
7.2 Item “Colostroterapia”	100	100	1	1
7.3 Item “Produção de leite materno”	100	100	1	1
7.4 Item “Ordenha do leite materno”	100	100	1	1
7.5 Item “Posição, oferta e mamada do recém-nascido	100	100	1	1
7.6 Item “Bicos e mamadeiras”	100	100	0,8	0,8
7.7 Item “Dispositivos para a amamentação”	100	100	1	1
Domínio 8 - Cuidados enquanto meu bebê está na UTI^*^ Neonatal				
8.1 Item “Cuidados enquanto meu bebê está na UTI^*^ Neonatal	100	100	1	1
Domínio 9 - Mamãe e papai, relaxem!				
9.1 Item “Mamãe e papai, relaxem!	100	100	1	1
Domínio 10 - Telefones e sites úteis				
10.1 Item “Telefones e sites úteis”	100	100	1	1
Domínio 11 - Referências				
11.1 Item “Referências”	100	100	1	1

* UTI - unidade de terapia intensiva;

** CPAP - continuous positive airway pressure.

* *UTI - unidade de terapia intensiva.*

Na segunda rodada, participaram cinco juízes, todos do sexo feminino, em sua maioria na faixa etária de 30-39 anos (60%), com o mestrado como formação acadêmica mais citada (40%) e área de atuação no âmbito assistencial por um período que variou de 10 a 20 anos (60%). A validação do conteúdo contou com a avaliação da cartilha como um todo, incluindo o título, o formato (layout) e as instruções de cada item separadamente, com base nos conceitos de clareza e pertinência/representatividade, em uma escala Likert de 1 a 4. O material manteve os 11 domínios na segunda rodada, e eles foram avaliados quanto à clareza e representatividade (Tabela 1).

Os juízes, na segunda rodada de validação de conteúdo, não alcançaram um IVC superior a 0,8 no item “Bebês e seu desenvolvimento”. A justificativa foi a necessidade de modificar a ilustração, pois poderia causar medo nos pais dos prematuros, além de não compreenderem seu significado. Sendo assim, optou-se por excluí-la da cartilha.

Além disso, mesmo com grau de concordância e IVC adequados, foram feitas outras edições com base nas sugestões dos juízes, como a mudança da cor da letra para melhorar a visualização, a alteração e a adição de palavras para aprimorar a especificidade dos itens e facilitar a compreensão dos pais. A validação final dos juízes atingiu um IVC global de 0,98. Após isso, a cartilha foi submetida à validação pelo público-alvo.

### Validação pelo público-alvo

Para a realização desta etapa, 30 participantes foram selecionados, sendo 28 (93,33%) do sexo feminino e 19 (63,33%) com idade entre 30 e 39 anos. Todos possuíam o ensino fundamental completo e 13 (43,33%) alegaram não ter conhecimento sobre prematuridade.

Os itens referentes ao conteúdo, nível de alfabetização, ilustrações, layout, estímulo, motivação, aprendizado e adequação cultural foram validados pelo público-alvo, uma vez que todos atingiram grau de aprovação igual ou superior a 80%. A avaliação geral da cartilha apresentou resultados muito positivos, com 95,39% das variáveis classificadas como “Muito bom”, enquanto o índice de reprovação foi de 0,95%. A maioria dos itens considerados inadequados estava relacionada à formatação da letra do texto (Tabela 2).

**Tabela 2:** Avaliação da Cartilha - Etapa de validação com o público-alvo, Porto Alegre, Rio Grande do Sul, Brasil, 2023

Variáveis	Muito bom (2) n (%)	Adequado (1) n (%)	Inadequado (0) n (%)
1 Conteúdo			
1.1 Quanto ao conteúdo da cartilha, você considera?	30 (100)	-	-
1.2 Você entende qual o propósito do material (fornece informações sobre a prematuridade e internação do bebê)?	30 (100)	-	-
1.3 A cartilha tem seu objetivo bem explícito e declarado no título e apresentação?	29 (96,67)	1 (3,33)	-
1.4 A cartilha foi útil com informações sobre a prematuridade e a internação do bebê na UTIN^*^?	29 (96,67)	1 (3,33)	-
1.5 A cartilha e seu conteúdo atendem ao objetivo (auxiliar com informações da UTIN^*^)?	29 (96,67)	1 (3,33)	-
1.6 A cartilha apresenta pontos-chaves, exemplos e ilustrações para melhor entendimento do texto?	30 (100)	-	-
2 Nível de alfabetização			
2.1 Quem leu a cartilha? (você leu sozinha (2)/em algumas partes precisou de ajuda (1)/alguém leu para você (0))	29 (96,67)	1 (3,33)	-
2.2 Sobre a escrita da cartilha?	29 (96,67)	1 (3,33)	-
2.3 Na cartilha é dado um contexto antes das informações?	28 (93,33)	2 (6,67)	-
2.4 As palavras utilizadas na cartilha são de fácil compreensão?	29 (96,67)	1 (3,33)	-
2.5 Há cabeçalhos ou legendas que informam brevemente o que vem a seguir?	28 (93,33)	1 (3,33)	1 (3,33)
3 Ilustrações			
3.1 Quanto às ilustrações, você acha:	28 (93,33)	2 (6,67)	-
3.2 As ilustrações têm relação com os conteúdos abordados?	30 (100)	-	-
4 Layout			
4.1 Há subtítulos na cartilha?	30 (100)	-	-
4.2 Quanto ao tamanho da letra da cartilha?	24 (80)	5 (16,67)	1 (3,33)
4.3 Quanto ao tipo de letra da cartilha?	25 (83,33)	3 (10)	2 (6,67)
4.4 Você identificou na cartilha: - O uso de cores não causa distração do conteúdo; - Que as ilustrações estão na mesma página do texto relacionado; - O layout e a ordem das informações apresentam uma sequência lógica; - Recursos como sombreado, caixas, setas são usados para direcionar atenção a pontos importantes; - As páginas são ordenadas.	30 (100)	-	-
5 Estimulação, motivação e aprendizado			
5.1 A cartilha te deu motivação para o enfrentamento da prematuridade e auxilia sobre atitudes e comportamentos?	27 (90)	2 (6,67)	1 (3,33)
5.2 Você acredita que as informações contidas na cartilha podem ser colocadas em prática - úteis?	30 (100)	-	-
6 Adequação cultural			
6.1 A cartilha auxilia com a sua experiência sobre prematuridade?	30 (100)	-	-
6.2 A cartilha aborda situações semelhantes às que estás vivenciando?	27 (90)	2 (6,67)	1 (3,33)

* *UTIN - unidade de terapia intensiva neonatal.*

Finalizadas as etapas de validação e de ajustes da cartilha, chegou-se ao resultado final do material. Assim, a cartilha educativa sobre prematuridade, intitulada “*Meu bebê prematuro na UTI Neonatal: cartilha para a família*”, é composta por 21 páginas, com capa, dimensão padrão de 21 cm de altura por 15 cm de comprimento, nas cores lilás e amarela. O conteúdo foi organizado em tópicos: A UTI Neonatal; Conhecendo meu bebê prematuro; Meu bebê precisou ficar na UTI Neonatal, o que eu preciso saber?; Amamentando um bebê prematuro; Cuidados enquanto meu bebê está internado na UTI Neonatal; Mamãe(s) e/ou Papai(s), relaxem! (Figura 1).

**Figura 1:** Capa, apresentação e sumário da cartilha educativa sobre prematuridade, Porto Alegre, Rio Grande do Sul, Brasil, 2023

## DISCUSSÃO

Os profissionais da saúde, principalmente os enfermeiros, desenvolvem a criatividade ao realizar o processo de cuidar e educar, fazendo uso de uma diversidade de tecnologias educativas^(29)^. Essas tecnologias devem ser empregadas de maneira que promovam a inclusão ativa dos indivíduos no contexto educacional, fortalecendo os valores de cidadania e autonomia entre os participantes^(30)^, auxiliando nos processos de ensino-aprendizagem e potencializando as ações de educação em saúde^(31)^. Dentre as tecnologias disponíveis na literatura para uso, como materiais informativos e educativos, destaca-se o uso de cartilhas educativas^(32-35)^.

Como tecnologia educativa, a produção de cartilhas tem papel importante na formação de conhecimentos e no desenvolvimento de habilidades de pacientes e/ou de seus familiares^(36)^. Assim, esse material contribui para a construção de um saber técnico-científico a partir de uma abordagem integrada das experiências dos profissionais e dos usuários, constituindo práticas sistematizadas, além de mediar o processo educativo e complementar as orientações fornecidas nos atendimentos de saúde^(37-39)^, ressignificando a experiência de hospitalização do neonato para a família.

Para transmitir as informações de maneira eficaz, é preciso atentar-se à escolha das palavras, utilizando um vocabulário prático para o público-alvo e criando um texto claro e de fácil compreensão^(40)^. Aliado a isso, o uso de cores, ilustrações e palavras em negrito torna a obra mais dinâmica, atraente e convidativa à leitura^(41)^. A cartilha sobre prematuridade trouxe um diferencial ao apresentar fotos reais que retratam a passagem do bebê prematuro pela UTIN, proporcionando aos familiares um primeiro contato com o ambiente e as rotinas empregadas de forma mais acolhedora e acessível. Esses elementos são importantes para a sensibilização das famílias e despertam o interesse pela leitura do material^(42)^.

Estudos que desenvolveram materiais educativos voltados ao tema da prematuridade revelaram resultados positivos em sua utilização, pois contribuíram significativamente para a construção do conhecimento e aumentaram a confiança das mães nos cuidados de seus filhos^(43)^. Além disso, promoveram o empoderamento dos cuidadores para a continuidade do cuidado e o preparo para a alta hospitalar de seus filhos^(32)^. Esses resultados corroboram a importância do desenvolvimento de tecnologias educativas que apoiem ações de educação em saúde para as famílias, especialmente em contextos de alta complexidade, como o cuidado neonatal^(32)^.

Nesse sentido, a literatura apresenta uma variedade de tecnologias amplamente utilizadas na educação em saúde pela enfermagem, desde as mais tradicionais até as mais inovadoras, como cursos online, workshops, rodas de conversa, aulas, discussões de casos, plataformas colaborativas, materiais didáticos, entre outros. Tais tecnologias desempenham um papel fundamental na promoção do cuidado centrado na família^(44)^.

Pensando nisso, as ações de educação em saúde mediadas pela cartilha educativa sobre prematuridade apresentam-se aos profissionais, especialmente à enfermagem, como uma ferramenta essencial para a prestação de assistência centrada na família, promovendo resultados práticos no cuidado e no entendimento da saúde do RN prematuro. Além disso, favorece o estabelecimento de diálogos e a criação de vínculos com as famílias, especialmente em momentos de grande vulnerabilidade emocional. Dessa forma, representa um avanço na melhoria da qualidade da assistência de enfermagem.

Vale destacar que, neste estudo, o material foi submetido à validação por especialistas da área, garantindo sua adequação técnica como etapa essencial. A etapa de validação por especialistas é fundamental para garantir a fundamentação científica do conteúdo apresentado, sendo imprescindível que os instrumentos utilizados para validar conteúdos de materiais educativos na área da saúde tenham a capacidade de abranger qualquer assunto com confiabilidade^(45,46)^. Dessa forma, a validação realizada por juízes-enfermeiros, tanto em termos de conteúdo quanto de representação visual, trouxe várias melhorias à cartilha sobre prematuridade, aproximando-a da experiência vivida por famílias que têm um bebê prematuro internado na UTIN. Além disso, a validação com o público-alvo também é necessária para garantir que o recurso atenda às necessidades específicas e seja eficaz no apoio às ações de educação em saúde.

Outro estudo que construiu e validou uma cartilha sobre prevenção de quedas no hospital, com especialistas, realizou a avaliação da adequação do material educativo com pacientes utilizando o SAM. Essa combinação estratégica torna o material mais robusto e fidedigno ao contexto dos indivíduos^(42)^.

A cartilha educativa validada neste estudo apresentou um IVC global de 0,98 pelos juízes e, pelo público-alvo, 95,39% das variáveis definidas como “Muito bom”, corroborando os achados da literatura que demonstram a relevância do tema discutido e a eficiência da tecnologia empregada na transmissão das informações^(6,42,47)^. Além disso, o material foi avaliado dentro de sua temática por especialistas da área de interesse, evidenciando a validade de seu conteúdo.

Destaca-se que essa cartilha se diferencia por ser voltada tanto para a preparação quanto para o período de internação na unidade neonatal, adotando uma perspectiva sensível e acolhedora para gestantes, puérperas e familiares^(42)^. Ela tem potencial para ser aplicada em diversos níveis de atenção à saúde em serviços da área materno-infantil, seja na Atenção Primária à Saúde, durante o pré-natal de risco para o parto prematuro, ou na própria UTIN dos hospitais, auxiliando nas orientações e no esclarecimento de dúvidas durante o processo de gestação de alto risco, trabalho de parto prematuro e nascimento prematuro.

### Limitações do estudo

Um fator limitante deste estudo foi o levantamento de temas sobre prematuridade realizado somente com profissionais da saúde que atuam na UTIN. A contribuição de usuários e de profissionais de outros níveis de atenção à saúde, como a Atenção Primária à Saúde, que acompanha o pré-natal das gestações com risco para o parto prematuro, teria enriquecido a discussão de possíveis temas para a construção da cartilha por meio de uma perspectiva diferente do cuidado à gestante.

### Contribuições para a área da saúde

O desenvolvimento de um material educativo, que aborda fatores de risco para a prematuridade e características do RN pré-termo, para gestantes e familiares de RN internados em uma UTIN, poderá auxiliar na promoção da saúde e na prevenção dos agravos ao neonato. Ademais, auxiliará na compreensão dessa fase de medos e inseguranças, ao proporcionar informações claras e apoio emocional para orientar as famílias a lidarem com os desafios da prematuridade. Além disso, trata-se de uma ferramenta facilitadora que pode ser utilizada pelos profissionais de saúde, especialmente os da enfermagem, para fornecer informações clínicas e orientações aos familiares de RN. Isso ajuda na preparação e no enfrentamento das adversidades associadas à prematuridade, garantindo a melhoria da qualidade da assistência à saúde na área materno-infantil.

## CONCLUSÕES

A construção e validação de uma tecnologia educativa em saúde, no formato de cartilha, visam preparar as famílias para os possíveis desfechos da prematuridade, além de ser uma ferramenta facilitadora para a melhoria da qualidade da assistência em enfermagem.

A participação dos juízes-enfermeiros contribuiu para a adequação e o fomento do material, que foi finalizado com IVC global de 0,98. Além disso, a participação do público-alvo, com índice de aprovação de 95,39%, demonstrou significância, confirmando o cumprimento dos objetivos propostos. O processo foi seguido criteriosamente, tendo como resultado um material representativo, didático e de fácil compreensão, ampliando a possibilidade de educação e promoção da saúde junto aos cuidadores de RN prematuros.

## Data Availability

Não se aplica.
